# Scientific Items

**Published:** 1877-08-20

**Authors:** 


### Scientific Items

MAN'S AVERAGE HEIGHT.—Statistical tables taken by army surgeons during the late war, show the mean height of over a half million men of different nativities. From these it appears that the American Indian stands at the head of the test, his mean height being 67.934 inches. The American white man is next in order, being 67.672 inches. The Scotch 67.066. English 66.575. Russia 66.393. France 66,227. Mexico 66.110.

A NEW MARINE DISTRESS SIGNAL.—A man in Vienna has devised a signal for the purpose of illuminating the sea when an attack by torpedo launches is possible. It consists of a shell containing a bottle full of a liquid capable of giving off phosphoretted hydrogen. Wet sponge surrounds the bottle, which is broken by the discharge from the gun; the liquid coming in contact with the wet sponge speedily generates gas, which drives out two stoppers, and two streams of illuminating matter are poured upon the sea.

TEMPERATURE OF THE INTERIOR OF THE EARTH.—From observations made on the Well of Sperenburg, near Berlin, M. Mohr concludes that at the depth of 5,170 feet the increment of heat must be nil. A similar decrease of the increment of heat has been observed in the Artesian Well of Grenelle. Hence, M. Mohr draws conclusions unfavorable to the Plutonian theory.—*Les Mondes.*

DAVYUM.– Herr S. Kerne of Russia, has discovered a new metal to which is given the above name in honor of Sir Humphry Davy. It has a silvery appearance; is extremely infusible, hard and to some extent ductile. Its specific gravity is 9.385.

